# Implementation of the PARTNERS model of care within two UK community mental health transformation systems: a qualitative realist evaluation

**DOI:** 10.1186/s12913-026-14959-4

**Published:** 2026-07-04

**Authors:** Jo Day, Julia Frost, Alex Stirzaker, Debra Richards, John Gibson, Vanessa Pinfold, Richard Byng

**Affiliations:** 1 NIHR Applied Research Collaboration South West, Department of Health and Community Sciences, Faculty of Health and Life Science, University of Exeter, Exeter, EX1 2LU UK; 2 Department of Health and Social Care, Faculty of Health and Life Science, University of Exeter, South Cloisters, University of Exeter, St Luke’s Campus, Heavitree Road, Exeter, EX1 2LU UK; 3 Community and Primary Care Research Group, University of Plymouth, ITTC Building, 1 Davy Road, Plymouth Science Park, Derriford, Plymouth PL6 8BX UK; 4 The McPin Foundation, Unit 1.4, The Green House, 244-254 Cambridge Heath Rd, London, E2 9DA UK

**Keywords:** Community mental health services, Consolidated Framework for Implementation Research, Implementation, Mental health transformation, Qualitative realist evaluation, Severe mental illness, Supervision, Leadership

## Abstract

**Introduction:**

PARTNERS is a model of care that was co-designed with service users and carers to address the needs of people with severe mental illness to receive better support in primary care. Taking learning from a randomised controlled trial and process evaluation of this complex person-centred goal and coaching-based approach to care, our aim was to understand better the challenges of implementing PARTNERS in complex dynamic service delivery systems.

**Methods:**

We identified two Integrated Care Systems that were interested in adopting the PARTNERS model of care. We trained practitioners to adopt PARTNERS to their local settings and provided meta-supervision (supervision of supervisors). We examined the implementation period that covered site engagement, training of staff and initial delivery of the new PARTNERS model of care, undertaking a qualitative realist evaluation informed by the Consolidated Framework for Implementation Research (CFIR). Data collection involved semi-structured interviews with 10 System Change Leads, Supervisors, and trained intervention practitioners or ‘Care Partners’; augmented by observations of supervision and practice within the systems and collation of documents. Analysis was qualitative informed by a realist approach and the CFIR.

**Results:**

Analysis identified complex overlapping configurations of teams and roles within them, leadership, and individual characteristics influenced the systems’ ability to implement the PARTNERS model of care. The presence or absence of leadership was instrumental in providing clarity regarding where the delivery of PARTNERS sat within newly emerging systems and was an important indicator of successful implementation. Collaborative leadership and supervision, alongside training tailored to the needs of individual practitioners, increased or decreased perceived self-efficacy amongst individual practitioners and their confidence in delivering the PARTNERS model of care.

**Conclusions:**

We identified that both internal and external supervision and system leadership are crucial to the implementation of PARTNERS, backed up by training delivered by a clinician and people with lived experience, to ensure that this new model of care is embedded in everyday practice. It is likely that the absence of any one of these mechanisms could make implementation and sustainability of the PARTNERS model challenging.

**Clinical trial number:**

Not applicable.

**Supplementary Information:**

The online version contains supplementary material available at 10.1186/s12913-026-14959-4.

## Background

The model of mental health care in England, and elsewhere, continues to evolve, with increasing emphasis on personalised care and global human rights [1]. The publication of the Community Mental Health Framework (CMHF) [2] for adults and older adults advocated integration of primary and secondary care for mental health, mental and physical health care, trauma informed care alongside elements of shared decision-making, coaching and social prescribing (NCCMH 2019) [2]. The CMHF proposed that care is provided in the community often across large disparate communities across Integrated Care System (ICS) areas by the right people, in the right place at the right time. While person-centred care was central to the policy, mechanisms for how it should be achieved were underspecified in policy papers [3, 4].

We were unable to identify any published peer-reviewed studies documenting the roll-out or specific aspects of the community mental health transformation in England. Guidance has been produced by Rethink Mental Illness [5, 6] and policy think tanks have written reviews of the opportunities afforded by structural changes including primary care networks and integrated care systems (hereafter ‘systems’) [7]. The framework also promotes a commitment to co-producing solutions with local communities and considering the role of diagnosis in a needs-led care system [8–11].

PARTNERS is a model of care co-designed with service users and carers to address the needs of people with severe mental illness to receive better support in primary care. PARTNERS is a recovery-based, collaborative care model developed using a realist approach to theory building [12] and refined during a formative evaluation [13].

The key mechanism of the model is a ‘Care Partner’ (envisaged as a mental health worker supported by a supervisor), located in primary care, working with a service user to build a relationship and develop a shared understanding, using a coaching approach to identify goals, linking with other practitioners and services as needed [15]. In PARTNERS, a training team consisting of service users and a clinician, provided initial training in model delivery for the Care Partners and supervisors. Top-up training was provided and reinforced with supervision and meta-supervision (supervision of the supervisors). The effectiveness and acceptability of PARTNERS was tested via a randomised controlled trial [15]. A process evaluation embedded within the intervention arm of the trial identified two key components of the model that were challenging for Care Partners to enact in practice: changing from ‘fixing’ to supporting the service user to identify individualised goals; and encouraging them to reach out to voluntary sector or additional primary care providers [16].

Research has identified that an organisation is best placed to assimilate an innovation more readily if it is large, mature, functionally differentiated and with concentrations of professional knowledge; if it has sufficient resource capacity to channel into new projects; and if it has de-centralised decision-making structures [17]. Additionally, researchers have conceptualised context as a dynamic system of agents with unpredictable and shifting relationships, rather than as a location, in which innovation requires continuous work, to both nurture and nourish accomplishments [18]. By considering the CMHF programme as a transactional space in which the community resources of the local system could be mobilised [19] we employed a qualitative realist evaluation informed by the Consolidated Framework for Implementation Research (CFIR) to guide our assessment of the contextual determinants helping and hindering the implementation process [20–22].

The delivery of the CMHF [2] provided an opportunity to examine the implementation of the PARTNERS model. In this paper we aim to develop further understanding of the challenges of implementing a complex person-centred goal- and coaching-based approach to care. This builds on our learning from a previous randomised controlled trial and process evaluation providing insights for implementation strategies/planning and implications for mental health service policy and transformation efforts [12–16, 23].

## Methods

### Study design

Consistent with guidance for developing and evaluating complex interventions [24], and building on our previous formative and summative evaluations [12, 13]^,^ we undertook a qualitative realist approach to the evaluation [25] of the implementation of PARTNERS informed by the domains of the CFIR [20]. We first outline the approach to implementation in the delivery systems and second the evaluation design/data collection.

### Collaborative optimisation

**Fig. 1:**
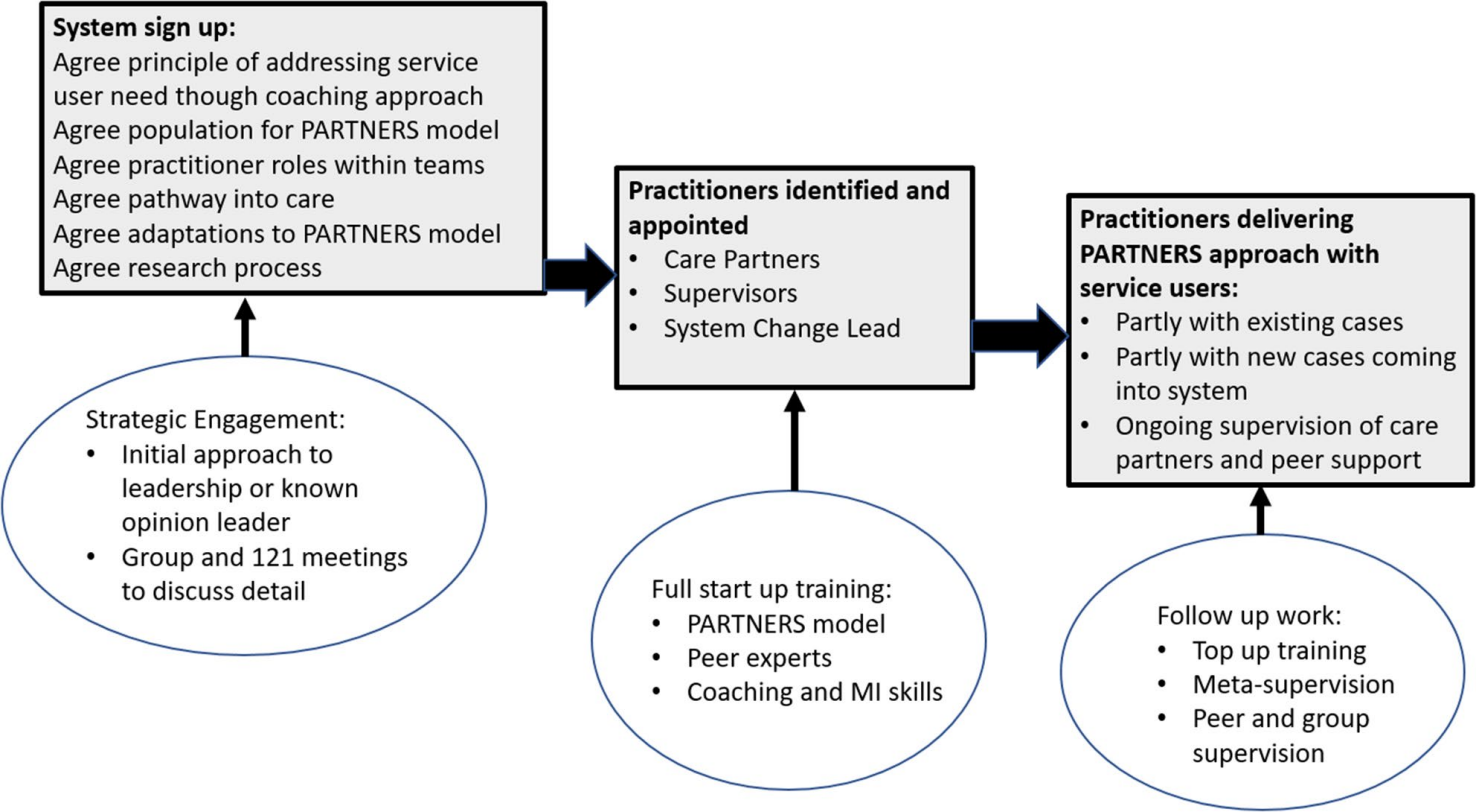
Collaborative optimisation: Pathway to implementation of PARTNERS model of care in new settings, supported by external research led delivery team

Our implementation approach was developed to apply learning from core components of the PARTNERS model of care [14] specifically paying attention to supervision, coaching approaches and integration of care, within two delivery systems who were part of the CMHF transformation effort. We termed the process of supporting the integration of PARTNERS into the two systems ‘collaborative optimisation’ (Fig. 1).

The opportunity to implement the PARTNERS model was advertised to all ‘systems’, by NHS England on our behalf, along with an information sheet about the intervention, which was distributed to commissioners and/or clinicians. [author] and [author] discussed with site representatives the viability of implementing PARTNERS within each system. After initial conversations, [author] and [author] identified which systems appeared likely to be able to deliver PARTNERS, being able to identify the client groups that PARTNERS could be used to support, e.g. those with lower motivation, psychosis, not high risk but potentially had not/or were not currently serviced well by existing services (e.g. people with stable psychosis who had limited contacts with services).

Once the two systems (here pseudonymised to protect their identity as: ‘Southtown’ and ‘Northside’), consisting of three ‘sites’ (‘Southtown’ and ‘Northside 1’ and ‘Northside 2’ ) were enrolled, members of the study team [authors] attended regular meetings with the systems about the implementation of PARTNERS. We supported systems to identify Care Partners to deliver, Supervisors and a System Change Lead to oversee and champion the implementation internally. A series of interactive training workshops tailored to the needs of each system were undertaken face to face and virtually over 2–3 days, in both locations, led by [author] and including members of the Lived Experience Advisory Panel (LEAP) from the study team, covering the programme theory and practical applications of the PARTNERS model. Training was supported by meta-supervision ([author] providing supervision to both Supervisors, and in the absence of a Supervisor, directly to Care Partners) and in-house Supervision (Supervisors provided group or individual supervision to Care Partners), and Peer Support (with Care Partners from within each system or site in attendance). Members of the study team attended training to share learning from previous PARTNERS evaluations, to support staff with administrative tasks associated with setting up implementation, and later, undertake observations, and to identify which staff to subsequently interview.

### Evaluation design and data collection

Our qualitative realist evaluation used interview, observational and document analysis methods and was informed by the Consolidated Framework for Implementation Research (CFIR) [21]. The approach was realist as we were interested in how the systems responded to implementation support to integrate the PARTNERS model into their transforming systems and we sought to identify the key implications for refining some of the programme theory propositions. The CFIR was chosen because it usefully identifies a broad range of known determinants influencing successful implementation. For this study we developed semi-structured interview schedules for each role in the implementation of PARTNERS, to explore challenges to implementation identified in our previous realist evaluations of the PARTNERS model [13, 16, 23] (Supplementary file: Example interview topic guide). These included the following two uncertainties identified from the programme theory :

1. Which mechanisms enable implementation of the PARTNERS model in the context of the community mental health transformation; and.
2. Which mechanisms enable team-based supervision, personalisation and coaching approaches to be integrated within the content of that transformation programme.

Interview questions were also informed by the CFIR domains/constructs [21] to enable exploration of existing practices and organisational cultures that may influence adoption and adaptation of the PARTNERS model [26]. We purposefully selected information-rich key personnel (staff with specialised, in-depth, or experiential knowledge)o participate in interviews, thus maximising the information power of our sample as we were aware they were not evenly spread across the three sites [27]. Verbal or written consent for participation was obtained from all of the participants in the study, as agreed with [REC]. Interviews with staff were undertaken by [author] and [author], and with participants’ consent audio-recorded and transcribed verbatim after being invited to take part and provided with an information sheet. Some participants preferred to not have their interview audio-recorded which, with their consent, [author] and [author] took annotated handwritten notes which were then transcribed. Alongside interview data, we collected documents and made reflexive fieldnotes/records from observations providing insights into the implementation process (supervision notes, site set-up/support visits, training/reflexive practice logs). Observation notes were structured using a template that included prompts to consider determinants from the five CFIR domains (intervention characteristics, outer setting, inner setting, characteristics of individuals and implementation process) influencing adoption/adaptation in each system. Informed consent was gained from participants for deidentified fieldnotes from observations to be taken.

### Data analysis

To facilitate our exploration of the systems and personnel, we constructed pen portraits by triangulating and synthesising our observations, interviews and documentary data to construct case descriptions at the level of practitioners, sites and systems – in line with the focus of the aims of our analysis [28].

These ‘thick descriptions’ [29] of the implementation context focused our attention on the evaluation of the PARTNERS model programme theory in situ [25] and this analysis was then developed further by focusing on the two uncertainties from the programme theory (noted above) and from the CFIR for identifying what influenced adoption of PARTNERS [21]. We then further focused specifically on areas found to pose challenges for implementation, e.g., causal pathways between supervision and clinical practices that enable personalisation and integration [16] which align with three of the CFIR domains: the implementation process, the inner setting (of each system) and the characteristics of individuals (practitioners and leaders). This led to further analytic attention to the role of key decision-makers and innovators which are also was drawn from CFIR. The final stage of analysis involved revisiting the two uncertainties from the RCT programme theory (noted above) for refinement.

The study team is multi-disciplinary with a range of backgrounds/interests including clinical practice, implementation science, complex mental health systems and lived experience of mental health needs, as well as experience in the voluntary sector, primary care and secondary mental health services and qualitative research. To make the analytical process accessible to members of the LEAP, analysis was undertaken through discussion, annotation and drawing the data, rather than by managing the data with computer software, with rigour ensured via [author] undertaking independent analysis, with discussion of tentative interpretations and triangulation of data sources with members of the wider research team to identify patterns within and across systems and sites [30].

Research Ethics Committee (REC) approval was provided by [University REC/ref number removed for peer review] and the study was approved by the two delivery systems.

## Results

The findings presented below present our understanding of how the PARTNERS model operated when implemented into two UK community mental health transformation systems, and the extent to which this enabled us to extend the programme theory underpinning the model. Throughout the study period, the implementation of PARTNERS in each system was impacted by staff absences/turnover, staff redeployment and COVID-19; which, in turn, impacted the data we collected (Table 1).

**Table 1:** Data collection

Practitioner *	Interview - audio recorded	Interview - annotated notes	Supervision field notes	Supervision record sheet	Meta-supervision field notes	Peer support field notes	CP reflective practice logs	System set-up Field notes	Practitioner (CP, SV) training Field notes
SCL1	1							✔	✔
SCL2								✔	
SCL3									
SCL4								✔	
SM1								✔	✔
SM2								✔	✔
SV1	1				✔			✔	✔
SV2								✔	✔
SV3	1		✔	✔	✔			✔	✔
SV4	1		✔	✔	✔				✔
CP1	2					✔	✔	✔	✔
CP2						✔	✔	✔	✔
CP3						✔		✔	✔
CP4						✔		✔	✔
CP5		2	✔	✔		✔	✔	✔	✔
CP6				✔		✔	✔	✔	✔
CP7		1	✔	✔		✔	✔	✔	✔
CP8		1		✔		✔	✔		✔
CP9	1		✔			✔		✔	✔
CP10						✔		✔	✔
CP11	1		✔			✔	✔	✔	✔
CP12			✔			✔		✔	✔
CP13						✔			✔
Total	8	4	7	6	3	13	7	19	20

**Key**: SCL = System Change Lead, SM = Service Manager, SV = Supervisor, CP = Care Partner

*Note: To prevent identification participants are not identified by delivery system/site

### Mechanisms enable implementation of the PARTNERS model

#### Implementation process: selection of individuals to deliver PARTNERS

**Fig. 2:**
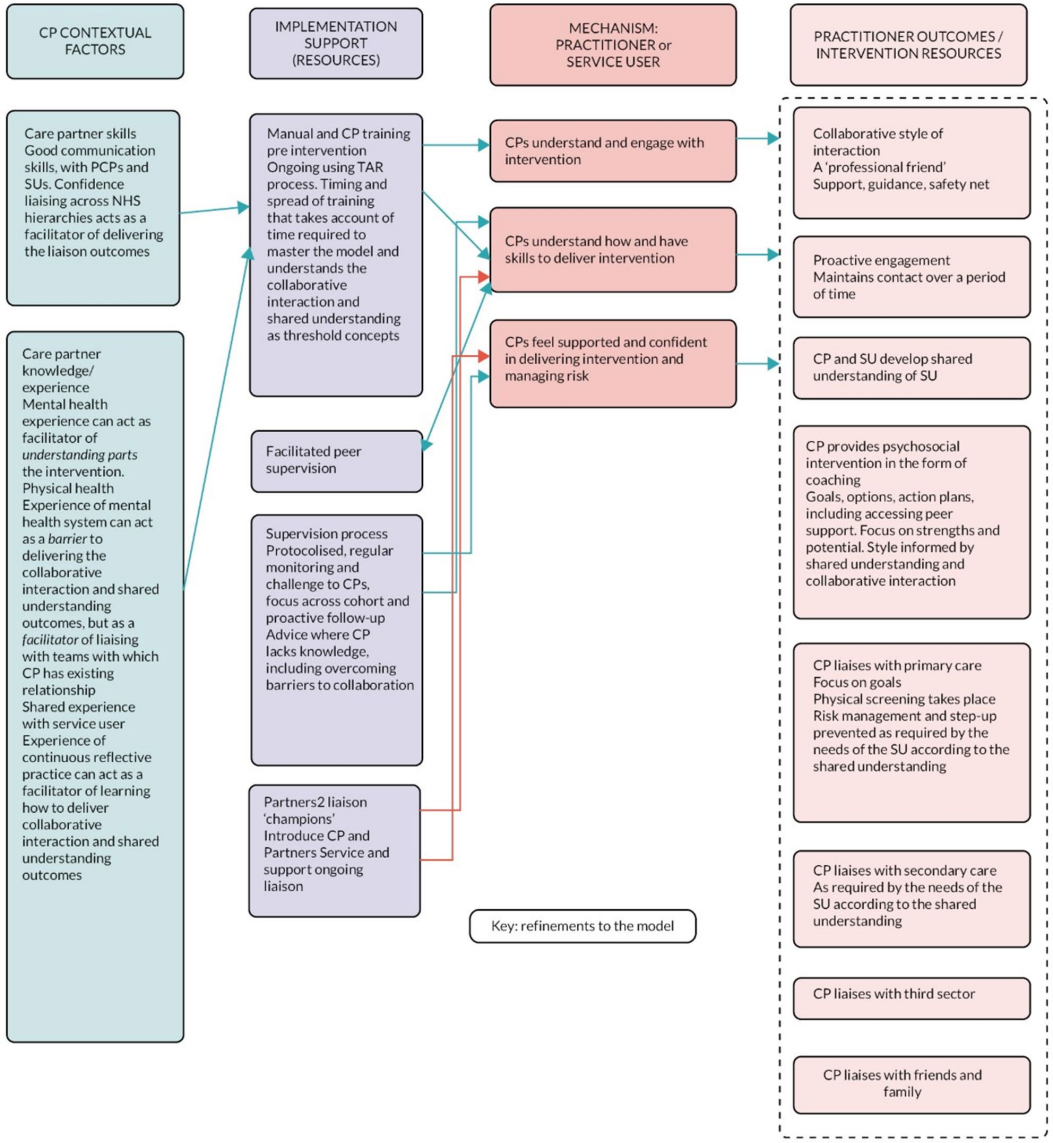
PARTNERS programme theory [14]

Initiation of PARTNERS delivery required a process of engagement by the research team with the system leaders (Fig. 2). Discussions took place with all systems, regarding alignment with the philosophy of the model and tension for change within the system, with [author] and [author] to co-produce training, and selection of Care Partners and Supervisors. As with the process evaluation from the main trial [16], individual characteristics of care partners and supervisors, such as their self-efficacy and readiness to adopt the model, were found to impact the attitudes and beliefs of their colleagues with respect to implementing the intervention.

##### Implementation process: champions and opinion leaders

In *Southtown*, discussions with both the System Change Lead and Care Partners identified Supervisor1 as a *Champion*, or implementation driver:

[F]rom what *CarePartner2* and *CarePartner1* have said, *Supervisor1*, just been really good at explaining the model and the approach because they’ve got that health coaching background and they’re an Occupational Therapist as well. … I remember *Supervisor1* saying when they first heard about the model, “none of this is new to me”, and … it sort of echoes how *Supervisor1* works anyway clinically some of this so it’s sort of turned out that they’ve been perfectly suited to this. As a supervisor they’re just really great at explaining and supporting and being passionate and enthusiastic and escalating where they need to do, being responsive. (*SystemChangeLead1* interview).

*Supervisor1* is amazing… I had a lot of anxieties when I first started… *Supervisor1* would just say: no, you’re doing this right…they provided me with some details on motivational interviewing. And just really made me reflect on things so when I was thinking I wasn’t doing something in maybe a way that I should have been, once I reflected on it, actually: yeah I am doing that. (*CarePartner1* interview2).

In the second day of PARTNERS training *Supervisor1* presented an adaptation of the PARTNERS model, which they successfully role played with one of the Care Partners, which exemplified the provision of individualised care.

Thus, in Southtown we recognised *Supervior1* as an effective and formal Champion who drove the implementation process, whereas in Northside 2 we found an Opinion Leader (*CarePartner5*), an informal influencer who supported implementation, and who wanted to adapt the model to suit their existing practice. *CarePartner5* was keen to develop a protocolised version of PARTNERS, which they suggested would validate their existing practice:

Noted similarities between PARTNERS approach and current practice. Quoted *CareParter5* as stating ‘This is how we do things’; also stated, ‘I don’t know how other model operates, but I know how *CarePartner5* does. (*CarePartner8* interview note).

*CarePartner5* also instigated site-led top-up training, after the period of formal training, which omitted motivational interviewing, an underpinning principle of the PARTNERS model.

Identifying the most appropriate people to engage in the implementation process is crucial to successful implementation.

#### Inner setting: structural characteristics

During initial PARTNERS implementation set up, both delivery systems were in the early stages of their transformation programme and still establishing new community facing services across their geographical areas. In *Southtown*, collaboration between the National Health Service (NHS) and Voluntary Community and Social Enterprise (VCSE) organisations was yet to be established, and the focus was on the development of the pathway between primary care and CMHT:

The Trust’s aspiration for the transformation programme is to collaborate with VCSE. However, they are not that far along in the process and need to investigate this further. (System Set-Up Fieldnote).

However, while there was already agreement on the need for pathways in primary, rather than secondary care, there were concerns about risk of harm, e.g. to a person’s mental health with the potential that risk would be elevated with inappropriate treatment, if non-professional VCSE or Band 4 workers [31] were deployed into the PARTNERS model:

So it was set up that all the service users that were transitioning out of CMHT to primary care mental health, only the band 6’s would work with that cohort of people so to try and get *CarePartner3* and *CarePartner4* to be able to work with those people has been a bit of a headache, because there’s worry about the risk and worry from partners, so the time that we’ve had to assure them and convince them, it’s just not been enough time…that hasn’t happened. (*SystemChangeLead1* interview).

In *Northside*, collaboration between the NHS and VCSEs had been partly established, but lack of access to electronic records fostered anxiety about clinical risk management. There was also a sense that practising PARTNERS care might conflict with normal NHS care, leading to questions about whether VCSE personnel taking up new roles in the system, could or even *should* take on the Care Partner role, engage with the implementation of PARTNERS, and the research processes:

[W]e’re a relatively new service, that it’s… it’s a relatively new partnership for the voluntary sector… and we’ve got a funny situation in *Northside 2* where our Care Partners can’t access… our electronic records even, so there’s some element of not being quite so cohesive as a team. Care Partners might… have a sense of, “Well, what if we’re not doing this correctly if… you know, according to NHS, according to the research team, according to…” so there’s something about having that confidence and that worry about whether they’re doing the right thing. (*Supervisor3* interview).

The absence of the System Change Lead at this point meant such anxieties could not easily be dispelled.

In both systems, small and localised teams were ‘volunteered’ to participate in the implementation of PARTNERS. In *Southtown*, mental health nurses were chosen because they were part of a small and stable working group, and a supervisor selected because of their experience and values regarding supervision and potential alignment to the PARTNERS model:

*CarePartner2*: Mental health nurse (community), previously a support worker. Liked that they’re involved in the process of change, rather than on the receiving end. Current caseload 30/35. *CarePartner1*: Newly qualified. Likes the evidence base of PARTNERS. Looking forward to working from a model. (Training Fieldnote).

So, coaching has always been something that I’ve been very, very interested in, and my manager knew that, and knows how much I value supervision and how much I promote the importance of supervision with… [they] felt that it was something that very much fit with how I already work, and [they] thought… [they] asked me to be involved [in PARTNERS] (*Supervisor1* interview).

In *Northfield*, some VCSE teams were selected by senior management, both to upskill non-qualified practitioners, and to enable a broad range of service users to be supported; while some Supervisors were selected from NHS partners because of their geographical location and experience:

It’s an unfortunate coincidence that PARTNERS landed with my new role as [practitioner] in the [another model] service in a system that is very inefficient. (*CarePartner8* interview).

The PARTNERS role was allocated to the geographical location where *Supervisor4* took up the post and was “a significant part of the role/job description” (*Supervisor4* interview note).

Thus, the social architecture in *Southtown* was suggestive of more stable inter-connection of teams, with more obvious functional differentiation between members. However, during the research period *Southtown* was not only managing the transformation project, but also dealing with the impacts of ‘business continuity’ meaning staff could be redeployed at short notice during the COVID pandemic:

Our… community mental health teams within our Trust, went into business continuity, Autumn last year, ….The whole Trust has come out of business continuity again, but CMHT remains in it. And during that time, I’ve been not formally redeployed, but essentially to all intents and purposes, redeployed into one of the community mental health teams, who have been particularly struggling with their staffing. (*Supervisor1* interview).

Despite these challenges, staff in *Southtown* were able to adapt the model to the needs of the service users who were already on their caseload, suggesting alignment with, and understanding of, the philosophy of the PARTNERS model:

My interpretation of the model is that it’s based really on health coaching with some sort of psychological understanding there. It’s aimed at people who have well maintained lower-level psychosis sort of symptoms so people perhaps with schizophrenia or bipolar, psychotic depression that kind of diagnosis, sounding a bit medical model there. So it’s an approach whereby, using an example of someone that’s maybe been attending a depot clinic for years in a few cases, where up until using this approach it was very much like, “here’s your injection, hope everything’s ok” and off they go. This is more about taking the time and opportunity to properly engage on their level and understand their story and understand what goals, if any, they would like to work on to try and improve their lived experience around their mental health and level of functioning and quality of life. (*SystemChangeLead1* interview).

In our initial meetings with *Northside 1*, the system change lead identified a clear pathway, via process mapping, for the recruitment and inclusion of service users into the implementation of the PARTNERS model. However, subsequent correspondence and meetings identified enduring confusion regarding which service users were most suitable for a PARTNERS approach:

*CarePartner9 and CareParter10* stated that they have been told to find service users with psychosis…Both have also been told service users they have identified… are not suitable. *CarePartner9* and *CarePartner10* are finding identifying service users with psychosis a barrier to starting. (Peer Support Meeting Fieldnote).

For me… it’s been confusing… I only really found out today that *Northside 1* had narrowed it down to a certain type of criteria because when I saw the email that *Researcher* had saying we’ve opened up and that’s what I assumed it was, so I’ve probably been giving *CarePartner9* and *CarePartner10* mixed messages. (Training Fieldnote).

The absence of the System Change Lead alongside, structural characteristics (as indicated by complex configurations of partner organisations, staff structures, and the extent to which a service user pathway could be identified), suggested that the current transformation climate added a further layer of complexity to the systems’ ability to implement the PARTNERS model.

### Mechanisms enable implementation of team-based supervision, personalisation and coaching approaches

#### Inner setting: networks and communications

In both systems, meta-supervision provided by [author], was valued, as a support framework for supervisors and as a safe space:

Well, I had regular supervision with [author]… and I think it benefitted the two people who I was supervising as well, I think knowing that I had regular opportunities to communicate with somebody who was involved in the project, and so that they were regularly being kept up to date with how things were… you know, a temperature check for how things were at our end. But also, to be able to then make sure that it was a two-way process in terms of feeding back (*Supervisor1* interview).

In addition, Supervisors across *Northside* valued meta-supervision as an opportunity to discuss pan-system challenges and share learning:

I think I’ve really found it helpful…when we were able to do it with Supervisor3 from *Northside 2* as well and getting a sense of where they’re at running parallel and I’ve really noticed that we’re in, we’ve certainly almost done things quite differently…That has been really helpful and I think just being able to talk about a lot, because I think for me a huge amount it has been system challenges, massively… I think I feel much more comfortable with the model, with the manual, with knowing what we should be doing…. [Meta-supervision] I think that’s been really helpful and just being able to talk through some of the difficulties I’ve had and just to be really validated. (*Supervisor4* interview).

I’ve been meeting with [author] for [meta]supervision, and with [Supervisor4] who’s the supervisor… from *Northside 1* And I guess that’s particularly helpful to hear what [SV4’s] doing, to have it in the partnership, because it… in the same way that group supervision hopefully is helpful for the Care Partners, has been helpful for me to kind of borrow ideas. And I guess it’s been quite helpful to reflect on what some of the barriers might be to implement and to make progress and think about that… (*Supervisor3* interview).

We additionally identified [authors]’ role - an ‘External Change Agent’ - as a key and perhaps an essential core component for implementation particularly in a transforming system.

Both systems opted to provide group supervision to Care Partners, which proved challenging. In *Southtown*, redeployment to more ‘essential’ work, and associated lack of capacity, inhibited its consistent delivery, but once Supervisor1 identified that the two Care Partners that they were supervising had different learning styles, they provided individual supervision:

The opportunities have been less than we’d hoped… But I think because [both Care Partners]… *CarePartner1* very much went with the, “I’m going to try and do everything to the letter, fill in every form, use every tool”, … They got started very quickly, because that was very much how they felt, if I don’t do it quick, I’m going to not know what to do. And so, *CarePartner2* felt very much like, “I’m in a different place”, and actually they were worrying that they weren’t doing enough. And it felt like they both that they needed something individually, so that they weren’t taking away from the other really. (*Supervisor1* interview).

Group supervision didn’t take place due to CarePartner1 and CarePartner2 lack of capacity to meet at the same time. CarePartner2 prefers 1:1 supervision. (Reflective Practice Fieldnote).

Because of the difficulty in identifying service users and starting to deliver the PARTNERS approach in *Northside*, here group supervision was taken up with more operational matters, such as familiarisation with the model and the study processes, rather than service-user facing reflections. There was also additional anxiety about the capability and confidence of some staff to work out how to flexibly deploy the different components of PARTNERS:

I think a lot of our supervisions to date have been massively about setting up, about challenges. Initially it was looking at the types of patients we could work with and initially with the Care Partners where they were struggling to identify the patients … I guess some of the logistics about identifying where would we see potential people? (*Supervisor4* interview).

So, there were points I think in supervision… I was feeling the pull to be kind of more directive than I might have wanted to be… And I think sometimes that was to do with the Care Partners not necessarily feeling confident enough about selecting something from the manual at that point… supervision might have a function in terms of supporting development for people, so supporting skills development as well as thinking about developing the confidence…a space to reflect and think about what’s working well… not working so well… I think particularly in group supervision, there’s something in particular for the Supervisor to think about, how do I make sure everybody’s involved, that nobody’s sitting on the side, that… and that everybody’s getting their needs met… It can be more complex in group supervision than it would be in an individual basis, when you’re trying to… keep on top of all those things… A Supervisor, it’s a bit like spinning plates. *(Supervisor3* interview).

Across both systems, peer support meetings provided an opportunity for shared learning amongst Care Partners. In *Southtown* participation in peer support with Care Partners from different teams enabled the cross-fertilisation of ideas across primary and secondary care:

I found *CarePartner3’s* knowledge invaluable … I just thought CarePartner3 had such a good outlook on workloads, on caseloads… I just thought they had such a lovely demeanour about them in person and I think they just had the right outlook. I think primary care were as struggling as much as secondary are with caseloads and workload but *CarePartner3* they put things into context for me… I thought that’s amazing, why have I not thought of that… (*CarePartner1* interview2).

Care Partners from *Northside 1* were more reliant upon the study team to manage and facilitate peer support sessions which tended to focus on matters such as the identification of service users.

We’ve done this in our last [peer support]… some of my colleagues that have already started in PARTNERS3, is sharing how it’s going for them…. we’re sort of concentrating on the SMI register and that, for them it’s slightly different as well, pathway. That might be really helpful to share. (*CarePartner9* interview).

Across the systems, meta-supervision, supervision and peer support provided new opportunities for discussing collaborative care across teams. Supervisors were typically able to understand better what the Care Partners needed to deliver the model and provided greater opportunities to develop reflective practice across the system as a whole.

#### Inner setting: implementation climate

Staff from across *Southtown* were receptive and welcoming of the PARTNERS model, recognising its potential for developing new ways of work with service users. In this case, the underpinning research evidence, the endorsement from individuals with lived experience, and the structured but flexible approach were considered important:

For me I need to have structure and know why I’m doing something and the reason for doing it… I was a bit apprehensive because I felt like it was a new thing that I had to learn, a new process and how am I gonna time this thing with everything else that I’ve got to do on a daily basis, but after having our training and the outcomes of other previous PARTNERS work, that you’ve done in the other areas and the evidence, what you’ve got on how it has worked but also listening to the people that are supporting the project, so [*Researcher]* and people like that who have their own lived experience, that for me resonated so because of [*Researcher]* involvement as well, that give me more incentive that yes it will work… … I need to have structure and I need to like put that into each visit… with the PARTNERS things, now I’m using that to sort of structure the goal setting element. (*CarePartner1* interview).

In the absence of the System Change Lead, staff in *Northside 1* were slower to implement PARTNERS, and we were only able to observe one Care Partner using the PARTNERS approach with one Service User:

*CarePartner11* felt their peers were looking to them for guidance because they had started implementation. They used the bullet points as a guide to following the model and feel confident in what they were doing. (*CarePartner11* interview note).

The implementation climate in *Northfield 2* demonstrated less capacity for absorbing and adapting to the PARTNERS model, within the timeframe of the study. In one of the VCSEs, a preference was expressed for working with a checklist or set of bullet points, rather than being guided explicitly by service user-identified goals. Receptivity to the PARTNERS model appeared to be moderated through the lens of one particularly influential Care Partner, who often described the implementation of the model in terms of ‘business as usual’, and sought to create a localised document for implementation:

*CarePartner11* was asked to share some bullet point notes from their first session as a structure of how they approached the new model, so that *CarePartner5* and others could add notes to make it a working document. (Peer Support Meeting Fieldnote).

*Supervisor3* endorsed *CarePartner5’s* suggestion of a list of questions. Described them as a list of open, gently curious questions’ that could be collected and listed for Care Partners to use. (Supervision Fieldnote).

In *Southtown*, we identified that meta-supervision nurtured a strong supervisory culture that ensured that, despite organisational challenges, Supervisors and Care Partners were supported in extending their practice. Staff in *Northfield* were more likely to defer to the research team to support the setting up of, and maintenance of, communicative networks, which then supplemented their existing modes of working.

#### Inner setting: leadership engagement

In terms of leadership, the *System Change Lead* in *Southtown* exemplified the values and ambition that underpinned the original PARTNERS model:

From a personal point of view I’ve never really been involved in research to this level, so for me it felt like a good objective for my appraisal. There’s a bit of personal gain there for my development, but also knowing what I knew about the community transformation ambition, it just sort of felt like, when I first heard about the research project and how it might impact clinically, it just sort of sounded a really great project to be involved with because it sort of rang true that population of people, that SMI population that sometimes go a little bit under the radar you know, I care about that cohort of people so it sort of you know it ticked lots of boxes in that respect. At the time I wasn’t sure how much work it would involve but yeah, I signed myself up for it anyway. (*SystemChangeLead1* interview).

Subsequently *SystemChangeLead1* was keen to share their positive experience of implementing the model, as a success despite specific parts of the organisation moving in and out of business continuity:

It’s been one of our very few good news stories over the last few months… I’ve been speaking to my manager about it, you know when it’s all doom and gloom, in our leadership meetings I’ve put my hand up and said or in clinical governance meetings and said, “actually can I just give you an update about how [PARTNERS] is going because this is something that we’ve managed to keep going and it’s really positive”, so I’ve just come from supervision with my manager who has supported me to support this project all along and they’re just really keen to engage with it and take it forward and celebrate it… and I think because it fits really well with community transformation, that really helps as well. (*SystemChangeLead1* interview).

In *Northside 1*, strong leadership was lost when the original *System Change Lead* left, and formal leadership taken over by a colleague who was new to the role and less involved in the PARTNERS project:

*Supervisor4* felt lacking support from a strategic point of view. Supervisor4 stated that as a leader without SystemChangeLead4’s support, worried about making the wrong decision. All the managers from the other organisations are turning to them. (Meta-Supervision Fieldnote).

Whereas in *Northside 2*, it was difficult to identify system leadership, with *SystemChangeLead2 (Feedback Event Fieldnote)* suggesting retrospectively, that they had not reached out to all the parts of the system as much as they would have liked to have done which was also noted below:

I’m just thinking maybe on reflection we could have been… informed others in the system a bit better about PARTNERS and the PARTNERS approach. …I’m wondering whether we could have done that in a better way or a kind of more comprehensive way and would that have made a difference to the Care Partners in trying to implement it. Because they were essentially trying to implement it in different areas across the trust that I… I didn’t necessarily have oversight of. So, I suppose in terms of a system, maybe there’s some support about that, about getting there and that information shared… (*Supervisor3* interview).

#### Characteristics of individuals: knowledge and beliefs about the PARTNERS model

We found that staff at all levels in *Southtown* were open to sharing their vulnerabilities with both the research team and the service users, with supervisors recognising Care Partners’ resilience in the face of adversity, e.g., the challenges of business continuity:

What I really liked was despite how clearly overwhelmed both staff were with the volume of work that they have…They were still looking for opportunities as to where they could use… the approach, and recognise that actually they were doing something different… there were ways of doing things which just meant that they understood that person better, or got a very different response with very little change to what they, as practitioners, needed to do. (*Supervisor1* interview).

In *Southtown*, both Care Partners had sufficient knowledgeable about the service users with whom they worked, and over the course of our research we were able to identify their readiness for change, which developed though their experience of implementing the model:

Able to step back and listen to the Service User and take a holistic approach and not assume to know or try to fix “I just sat there and listened to them and gave them the time”. Able to reflect on practice and recognise adaptations and changes to practice which are more service user orientated and able to draw on previous experience “I did it on the wards”. (Reflective Practice Fieldnote).

We also observed that the Care Partners in *Southtown* noted a perceived strength in sharing their authenticity with the service users with whom they were working:

A lot of the Service Users I’ve said: “Look we’re going to look at it in a different”, and with that Service Users in particular, he were like “good, because I don’t think the way we were working before has helped me at all.” … Why’s he never been given this? Why’s someone not asked him what he’d like to do, goal setting for his future? Why was the workplace stuff not implemented? Maybe that because he just wasn’t at that stage before. (*CarePartner1* interview2).

In contrast, some of the staff in *Northfield 2* were perceived as being uncomfortable in sharing their professional vulnerability and were worried that they might not be able to get things ‘right’. Therefore, staff were less comfortable sharing with service users that they were trying a new method of practice:

*CarePartner5* felt unable to talk to the service user about using a new way of working or inviting to take part in the study – service user might feel it’s an experiment. *CarePartner5* felt it was not right to mention the model to the Service User when they text them positive feedback about their interaction and how they had found the experience different. (Peer Support Meeting Fieldnote).

*CarePartner7* didn’t tell service users they were taking a new PARTNERS approach as felt they might feel they were ‘a guinea pig’. (*CarePartner7* interview note).

While Care Partners in *Southtown* understood the principles and rationale for adopting the PARTNERS model for use with the service users with whom they worked, and share this learning explicitly with those service users, Care Partners from *Northfield* were more likely to resist the adoption of a new way of working with service users, preferring not to disclose that they were trying new principles or practices.

#### Characteristics of individuals: self-efficacy

Senior staff in *Southtown* identified and recognised their agency and capabilities to promote the model, including the reflective style of supervision, and also that they were able to sit with uncertainty. Speaking about supervision:

I know so many people who have been put off from engaging in supervision, because their experience has been that they go and they get told what to do, and they’ve been told what they’ve not done, and it doesn’t feel like an experience that has helped them to grow… When I talk to people about what you want from supervision, I also ask what you don’t want…Because sometimes people are more able to articulate what they don’t want from it… I don’t think you always have to be highly knowledgeable in a specific area, because part of the skill of being a supervisor is to say; well, I don’t know either…But how can we find out. That also links in with coaching as well, but in terms of… yeah, not setting yourself up as an expert in everything. (*SystemChangeLead1* interview).

[PARTNERS model] was something that really made a lot of sense to me, it really was sort of aligned with my values as an individual… So, coaching has always been something that I’ve been very, very interested in, and my manager knew that, and she also…Knows how much I value supervision and how much I promote the importance of supervision with other staff… … There was a lot more opportunity for having a go and being observed having a go and getting feedback. (*Supervisor1* interview).

In *Northfield 2*, with the loss of the *System Change Lead* early in the research period, and where the *Service Manager* described themselves as ‘overseeing from a far’ (Problem-solving meeting fieldnote), we identified that less experienced staff and those new to mental healthcare were less confident in their ability to make the changes required to implement PARTNERS:

*CarePartner8* regarded PARTNERS manual as okay but a ‘way to shrink it would be epic for newbies’. Mentioned protocolising PARTNERS through mini-manuals or checklists/checkpoints, with lists of questions. Suggested maybe using a short (5-minute) cartoon or video as part of training or for Care Partner role…Thought that the PARTNERS training should be an accredited course. (*CarePartner8* interview note).

A noticeable exception in this system was *CarePartner11*, who had more experiential knowledge:

*CarePartner11* has experience of working on pilot models which has helped them to be flexible and “get stuck in” “Giving it a go” feels familiar. Although, they still feel they needed more structured guidance “having a very structured, simplified template to work to I think is incredibly helpful”. (*CarePartner11* interview note).

Individuals in *Northside* were generally reluctant to embrace the full potential of PARTNERS, instead proffering a more bite-size version that required minimal changes to their current context or actions. This contrasted with individuals in *Southtown*, who demonstrated higher levels of self-efficacy, and could consistently identify when and how a PARTNERS approach would enable staff to achieve their potential.

## Discussion

We undertook a study to implement and evaluate the PARTNERS model using a qualitative realist approach, informed by CIFR three domains/constructs – the ‘Inner Setting’, ‘Characteristics of individuals’ and ‘Implementation process’ [20]. We further developed our understanding of the key mechanisms helping and hindering it’s use to better support future implementation and refined two aspects of the PARTNERS programme theory. We identified that in the context of stressed systems undergoing a wider transformation programme, both external and internal supervisory practices were necessary for initiating the implementation of PARTNERS, and that both core organisational (‘Inner Setting’) and individual values (‘Characteristics of Individuals’) need to align with the ethos of the PARTNERS model [15]. This requires robust and high-level ‘Leadership Engagement’ and support to navigate the changes required to integrate the PARTNERS model within actively transforming delivery systems.

In both systems meta-supervision, in this case provided by an external clinician, was well received and valued. Meta-supervision enabled shared learning between the supervisors from *Northside 1 and 2*; however the group supervision of Care Partners in both of these sites focused on operational and administrative matters. Previous research has suggested that the content of clinical supervision in community mental healthcare can be subsumed by administrative tasks when supervisors lack experience and when organisations are less familiar with implementing evidence-based initiatives [30] and do not have the benefit of strong system leadership to advise on implementation. In contrast, in *Southtown* we identified an internal System Change Lead (‘Champion’) who fostered an ‘Implementation Climate’ that enabled the Supervisor to tailor supervision to the individual Care Partner’s needs. This builds on research which has identified that the mechanisms of supervision enabling innovation are the diffusion and synthesis of information (tailored to the needs of staff), mediation between expectations and actions (via open communication and monitoring), and selling the implementation, which together support the adoption of the initiative as intended [33].

In *Southtown*, we identified that despite being in ‘business continuity’, which diminished both the staff capacity and time available (‘Inner Setting’), with continued support from Supervisors and System Change Leads who fully endorsed the use of the model, some Care Partners were able to implement a PARTNERS approach with service users, and report a positive impact on both service delivery and service user outcomes (e.g. personalised goal setting) within a short time frame. In Southtown, the System Change Lead acted as a ‘Champion’ for the implementation of PARTNERS, while Care Partners demonstrated both resilience in terms of challenges within the system and a level of skill and experience (‘Characteristics of Individuals’) that fostered a readiness for change in order to support the service users with whom they were working. Implementation was slower in *Northside 1*, and in *Northside 2*, where the loss of the System Change Lead (‘Champion’) during the implementation process impacted on the confidence of both Supervisors and Care Partners to identify a service user population with whom to work, and where it was proposed that the adaptation of the PARTNERS model be reduced to a set of questions or checklist that could be used with all service users. In the absence of a ‘Champion’, we identified an ‘Opinion Leader’ (a Care Partner in *Northside*), whose strong views about the structure, rather than ethos of the PARTNERS model, influenced how other Care Partners viewed and talked about the implementation and adaption of PARTNERS. This reflects research which identified that staff with less confidence are less able to turn learning into meaningful engagement, and more inclined to prefer standardised approaches that may be less suited to implementing innovations, in our case, through delivery of person-centred approaches [34].

System Change Leads (‘Champions’) in this study were particularly instrumental in supporting and enabling change in the stressed delivery systems. Giving permission to Care Partners and Supervisors to try something different, by offering support with decision making was crucial for implementation to be successful. This resonates with existing research that highlighted how making changes can seem destabilising, leading to mixed responses amongst care providers particularly during a period of large-scale change (‘Inner Setting’). This can lead to potential challenges that may occur when care providers become disengaged from the change process – viewing risk as a reason not to implement an initiative and to retain existing or revert to old practices [35]. The presence or absence of a System Change Lead (‘Champion’) was therefore a key mechanism in enabling or hindering the implementation of PARTNERS in transforming delivery systems. Our learning from this complex transformation landscape is that some systems/sites may require a greater degree of support scaffolding (e.g. a greater dose of training and ongoing external support) to ensure that change within the existing architecture is sustained [32].

Our findings augment PARTNERS realist programme theory [15], enabling two aspects to be further refined.

### *If* supervisors can be identified/supported by an internal change agent then supervisor can empower care partners to employ the partners approach and build their confidence to approach working with service users with openness

In contrast, where Supervisors allowed the content of supervision to focus on operational and administrative matters, Care Partners are reluctant to implement for fear of making mistakes. This was influenced further by the absence of a leader who can give permission to try new ways of working and allow for mistakes to be made in a safe context. The absence of the system change lead (‘Champion’) added to the lack of clarity regarding the identification of appropriate service users hindering implementation.

### *If *care partner’s values align with the PARTNERS model, and an appropriate service user population and their goals can be identified, *then *care can be personalised

In contrast, where Systems failed to identify a Service User population, and sought to implement PARTNERS within the confines of their existing organisational model (typified as ‘business as usual’), we did not observe care becoming more personalised.

**Fig. 3:**
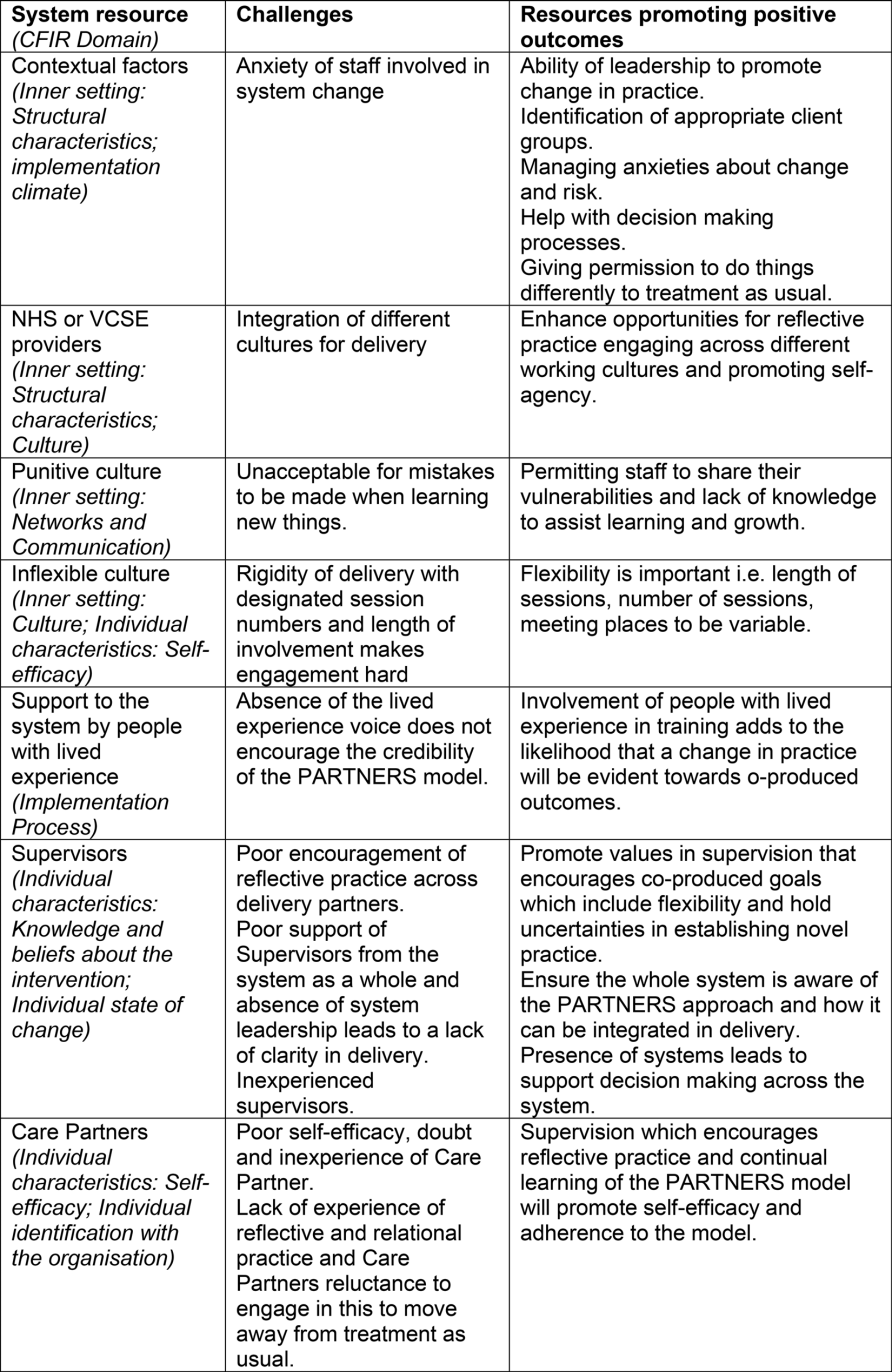
Resources required for the implementation of the PARTNERS model

Work is underway to prepare the PARTNERS model for wider use, and key areas for future focus are how best to (1) include local service users in any training, (2) engage with leaders of service change and (3) identifying how best to align the meta-supervision, supervision and peer support structures to local systems which are appropriately supported by system leadership to enable implementation. The findings from this provide recommendations of the resources necessary to support implementation (Fig. 3) informed by CFIR, which may also be applicable to the implementation of other, similar evidence-informed models of care being integrated into complex dynamic community health and social care settings.

A strength of this research is that we were implementing a previously tested theory-informed model and training package [14, 16]. An additional strength is that we identified and trained staff to deliver the PARTNERS approach within a relatively short timeframe in the real-world context of the community mental health transformation programme. The use of CFIR was valuable for enabling us to unpack what was influencing the implementation of PARTNERS – particularly the context of the inner setting of each delivery system, the process of implementation and the importance of individual roles and characteristics. However, implementation and evaluation was hindered by the requirements of central and local research ethics and delivery processes, which inhibited our ability to support the systems to implement their learning in a timely manner (the perceived burden both implementing and evaluating placed on systems/sites during a time of transformation), in particular delaying data collection in one site. There is a need for proportional and responsive ethics and governance process for implementation research. Due to time constraints, we were unable to incorporate the perspectives of individual service users and were reliant upon the perspectives of the Care Partners who support them. Further feedback is required to explore the perspectives of Service Users and their carers on the benefits of the implemented PARTNERS model.

## Conclusion

Having previously developed and refined the programme theory that underpins the PARTNERS model, and despite the difficulties in the system delivery and research landscape in the UK at the time of implementation, we developed our understanding of implementation planning and gained insights to inform future mental health policy and transformation efforts. We identified that attention to the inner setting of systems (particularly implementation climate) and characteristics of individuals involved in the implementation (leaders and practitioners), are fundamental to both successful implementation and the development of policy implementation guidance. System leadership and supervision are crucial to enabling the implementation of the PARTNERS model – which has enabled us to refine two propositions of the PARTNERS programme theory. We identified that our implementation strategy, and training and supervisory support, needs further refinement to meet local system delivery needs.

## Supplementary Information

Below is the link to the electronic supplementary material.

Supplementary Material 1

## Data Availability

Transcripts will not be shared in their entirety to protect the anonymity of participants and workers delivering the interventions. However, requests for excerpts of the data will be considered on an individual basis. Please contact the corresponding author.
