# Supplementary material for: Implementation of the PARTNERS model of care within two UK community mental health transformation systems: a qualitative realist evaluation
Source: BMC Health Serv Res. 2026 Jul 4;26:931. doi: 10.1186/s12913-026-14959-4 (PMC13340360; doi:10.1186/s12913-026-14959-4)
Supplement: Supplementary file 1 — Supplementary Material 1 [file 12913_2026_14959_MOESM1_ESM.docx]

**Supplementary File: Example Interview Topic Guide**

**PARTNERS3 Implementation Study: Care Partner interview topic guide**

For all areas of questioning, try and elicit specific examples, using probes such as:

• *Can you give me an example of that?*

*• Can you tell me about a particular time that happened?*

*• What happened that made you think that?*

**Start of interview:**

Explain the purpose of the interview:

- We want to find out how the intervention works in practice and what circumstances are needed for it to work best
- We will do this by going through the different aspects of the PARTNERS service and how much the service has worked the way we expected it to work (the programme theory).
- We are not evaluating performance and will not feedback your comments to other practitioners or researchers outside the qualitative research team.

The interview should take about 60 to 90 minutes

**Part 1: Context**

| 1.1 Could you tell me about your experience as a mental health practitioner prior to becoming involved in implementing the PARTNERS model?  *Prompt for:*   - *Role (e.g., nurse, social worker)* - *Service settings (e.g., CMHT, in-patient, primary care)* - *Length of mental health experience* - *Previous experience of coaching or similar psychosocial interventions* |
| --- |
| 1.2. What led you to become part of the study? What were your expectations of it at the start?  *Prompt for:*   - *Hopes or expectations,* - *Understanding of the nature of the intervention including collaborative care and personal recovery* - *How much choice they had in joining PARTNERS – for example, were they volunteered?* |

**PART 2: Becoming familiar with the PARTNERS model**

| **2.1. What were your impressions of the PARTNERS intervention (during and after initial training) & how similar/different is it to previous ways of working? What are the similarities with/differences to the MHICS and GPiMHS models? In terms of approach, timescales, skillset required. Could you tell me about your experience of finding suitable service users?**  *Prompt for:*   - *How comfortable and confident do they now feel about the model?* - *Anything that prevented or delayed starting to implement it* *Explore any sense that there have been stages to their learning about what PARTNERS involves, and/or that their understanding of PARTNERS has changed over time – if so, what brought about the development in their understanding?* |
| --- |
| **2.2. Could you talk about your early experiences of putting the PARTNERS intervention into practice? How easy/difficult has it been to incorporate into your practice? What do you understand by the term ‘business as usual’?**  *Prompt for:*   - *What’s been easiest/most challenging?* - *Anything not sure about?* - *Have you come to understand how to do/get around this, and if so, how?* |
| **2.3. How have you found the range of supports provided to learn about PARTNERS? Were some more useful than others?**  *Prompt for:*   - *Initial training – which aspects? the role plays? the lived experience element to it?* - *Use of the Care Partner manual* - *Contact with members of the research team* - *Other relevant reading or training that has been accessed (e.g., on coaching)* - *Anything else* |
| **2.4 What is your experience of supervision? What do think the value of supervision is?**  *Prompt for:*   - *How often supervision taking place* - *Examples of advice given* - *Relationship with supervisor* - *Use of protocol* - *Helpful aspects or challenges of supervision* |
| **2.5 How have you been finding peer support? What do you think the value of peer support is?**  *Prompt for:*   - *How often peer support is taking place* - *Examples of content of these meetings and how useful it has been* |
| **2.6 What qualities or attributes make a good Care Partner? Could you tell me why do you think that?** |

**PART 3: Sessions with service users**

| **3.1 Could you tell me about starting to build a collaborative relationship?**  **What’s worked well, any challenges, why? And how these have been overcome**  *Prompt for:*   - *How they would characterise a collaborative relationship* - *What s/he did to try and achieve this* - *How service users responded* - *Any effect on service users’ health or wellbeing, and whether a non-hierarchical relationship of equals made it easier for service users to be honest about their wishes to change their life/lifestyle habits/difficulties working towards goals* |
| --- |
| **3.2 Can I ask you about developing a shared understanding?**  **What’s worked well? Have there been any challenges? If so, why? Have you been able to overcome these? If so, how? If not, can you reflect on why?**  *Prompt for:*   - *How they would characterise a shared understanding* - *What s/he did to try and achieve this* - *Any resources used* - *How service users responded* - *Any effect on service users’ health or wellbeing* - *The extent to which understanding the individual service user means they are able to support the person to identify and work towards goals* |
| **3.3 What is it like using a coaching approach to setting goals and action planning?**  **What’s worked well, any challenges, why? And how these have been overcome**  *Prompt for:*   - *How they would characterise coaching* - *How s/he did the four stages (identifying and prioritising goals, exploring realistic approaches to working towards goals, discussing strategies and resources to draw on, creating a shared action plan)* - *Any resources used* - *How service users responded* - *Any effect on service users’ health or wellbeing, including the way they perceived themselves and their ability to make changes within their lives, and/or any action they are able to take* |
| **3.4 Could you tell me about whether you’ve been able to review the service user’s progress, both towards his/her goals and of his/her general wellbeing?**  **What’s worked well, any challenges, why? And how these have been overcome**  *Prompt for:*   - *How the Care Partner did this (e.g., motivating service users towards change, adapting shared action plans and goals to be more realistic/meaningful, supported to cope with successes and setbacks, stability of MH)* - *Any resources used* - *How service users responded* - *Any effect on service users’ health or wellbeing, including the way they perceived themselves and their ability to make changes within their lives, and/or any action they are able to take* - *Reasons s/he thinks it might or might not have worked well* - *Any effect on service users’ health or wellbeing this may have had* |
| **3.5 Have you been pro-actively following up of any service users who have fallen out of contact? How have you found this approach?**  **What’s worked well, any challenges, why? And how these have been overcome**  *Prompt for:*   - *How s/he provided consistent contact over an extended period of time, including coping with setbacks to appointments* - *How the service user responded* - *Any effect on the service user’s wellbeing* |

**PART 4: Aspects of the Partners service involving liaison with others to support the service user**

| **4.1 Have you been able to liaise with other agencies (including primary care, secondary care mental health, third sector, community organisations)**  **What’s worked well, have there been any challenges - why? Have you been able to overcome these? If so, how?**  *Prompt for:*   - *Who have they liaised with? Including signposting, referrals* - *Did they communicate any aspects of the shared understanding of a service user (with SU’s consent) to primary and secondary care mental health workers/third sector/community organisations? If so, do they think this supported more appropriate provision to the service user by the practitioner/third sector/community organisation?* |
| --- |
| **4.2 Have you involved carers, family members or friends in the care of the service users you have been seeing?**  **What’s worked well, have there been any challenges - why? Have you been able to overcome these? If so, how?**  *Prompt for:*   - *Service user interest in involving family or friends,* - *Did they communicate any aspects of the shared understanding of a service user (with SU’s consent) to family/friends?* - *Responses of family and friends, particularly any development of coaching attitudes/skills that may have improved support to the service user* |

**PART 5: Any other observations**

| **5.1 Could you tell me about any effects of the intervention you’ve so far observed on service users’ mental or physical health, quality of life or personal recovery?**  *Prompt for: what specifically they think has brought about these effects* |
| --- |
| **5.2 Do you have anything else to feed back about the intervention?**  *Prompt for:*   - *What they like most about the service, what they find most challenging* - *Was there anything they expected you to ask that you haven’t asked?* |
